# Sex-Specific Associations Between Physical Activity, LDL Cholesterol, and Functional Performance in a Real-World Primary Care Population: A Cross-Sectional Study

**DOI:** 10.3390/jfmk11030254

**Published:** 2026-06-27

**Authors:** Peter Marián Kalanin

**Affiliations:** 1 Institute of General Medicine, Faculty of Medicine, Pavol Jozef Šafárik University in Košice and MED-KAL, s.r.o., 040 01 Košice, Slovakia; peter.kalanin@upjs.sk; 2 Institute of Physical Education and Sport, Pavol Jozef Šafárik University in Košice, 040 11 Košice, Slovakia

**Keywords:** physical activity, sex factors, cholesterol, LDL, primary health care, mobility limitation, Timed Up and Go test, cardiometabolic risk, cross-sectional study

## Abstract

**Background**: Physical activity (PA) is associated with cardiometabolic health and functional performance, yet sex-specific evidence from real-world primary care populations remains limited. **Objective**: This study evaluated whether associations between self-reported PA categories and LDL-C concentrations and Timed Up and Go (TUG) functional performance differed between males and females in a real-world primary care cohort. **Methods**: This cross-sectional observational study included 863 adult primary care patients (424 males, 439 females). PA was categorized as low, moderate, or high based on World Health Organization recommendations. LDL-C and TUG were assessed as primary outcomes. Sex-stratified analyses included one-way ANOVA with Bonferroni post hoc comparisons and multivariable linear regression adjusted for age, BMI, arterial hypertension (AH), and diabetes mellitus (DM). An interaction term (PA × sex) was included to formally test for sex modification. **Results**: Males and females did not differ significantly in age, BMI, LDL-C, TUG, AH prevalence, DM prevalence, or PA distribution (all *p* > 0.05). Higher PA categories were associated with lower LDL-C in both males (ANOVA F = 13.03, *p* < 0.001) and females (ANOVA F = 14.92, *p* < 0.001), and with better TUG performance in both males (F = 44.21, *p* < 0.001) and females (F = 36.09, *p* < 0.001). In multivariable regression, PA was the strongest independent predictor of both LDL-C (males: β = −0.301, *p* < 0.001; females: β = −0.323, *p* < 0.001) and TUG performance (males: β = −1.235, *p* < 0.001; females: β = −1.170, *p* < 0.001) in both sexes. The interaction term (PA × sex) was not statistically significant for either LDL-C (*p* = 0.804) or TUG (*p* = 0.944), indicating no significant sex modification of the PA–outcome associations. **Conclusions**: The associations between self-reported PA and both LDL-C concentrations and TUG functional performance were consistent across sexes in this real-world primary care cohort. These findings support the clinical relevance of routine PA assessment as a sex-independent indicator of cardiometabolic risk and functional health in primary care. Because of the cross-sectional design, causality cannot be established.

## 1. Introduction

Physical activity (PA) is a central determinant of cardiometabolic health and functional capacity [1,2]. A substantial body of evidence demonstrates that higher PA levels are associated with more favorable lipid profiles, lower cardiovascular risk, and better functional performance across diverse populations [3,4,5]. In primary care settings, routine PA assessment may provide clinically useful information about cardiometabolic risk status and functional health that complements conventional biomarkers.

Sex-related differences in cardiometabolic risk factor profiles, lipid metabolism, and functional performance are well recognized [6,7]. Women consistently demonstrate higher levels of physical inactivity globally, and the cardiovascular consequences of inactivity may differ between sexes due to hormonal, physiological, and behavioral factors [8,9]. Despite this, evidence specifically examining whether associations between PA and cardiometabolic outcomes are modified by sex in real-world primary care populations remains limited.

The biological basis for considering sex as a potential effect modifier in exercise-related research is well established. Male and female skeletal muscle differ in contractile properties, fiber-type composition, fatigue resistance, and anabolic hormonal milieu [10]. These morphological and functional differences underlie documented sex-related divergences in neuromuscular performance characteristics and responses to exercise training [11]. It remains unclear, however, whether such intrinsic physiological differences translate into meaningful sex modification of the associations between habitual physical activity and cardiometabolic or functional outcomes at the population level—a question with direct clinical relevance for primary care practice.

Previous reports from the present cohort demonstrated significant associations between self-reported PA and LDL-C concentrations [12], arterial hypertension [13], and type 2 diabetes mellitus [14] in an unselected primary care population. However, these analyses did not formally examine whether these associations differed between males and females or assess the potential sex-modifying effect of PA on cardiometabolic and functional outcomes.

Understanding whether PA–outcome associations are consistent across sexes is clinically relevant for primary care. If associations are similar in magnitude and direction in both sexes, routine PA assessment can be recommended as a sex-independent clinical tool. If significant sex modification exists, sex-specific approaches to PA screening and lifestyle counseling may be warranted.

Therefore, the primary aim of the present study was to evaluate sex-stratified associations between self-reported PA categories and LDL-C concentrations and TUG functional performance in a real-world primary care cohort, and to formally test whether sex modified these associations. We hypothesized that PA would be associated with both outcomes in males and females independently, with no significant sex modification.

## 2. Materials and Methods

### 2.1. Study Design and Setting

This study was designed as a cross-sectional observational analysis using routinely collected clinical data from a single primary care practice in Košice, Slovakia. The analytical database comprised de-identified electronic medical records extracted systematically from consecutive patient contacts over the study period (February 2021–May 2026). The database is not publicly registered, as it consists of fully anonymized routine clinical data rather than a prospectively designed research registry; the dataset was closed at the end of May 2026 and statistical analysis was completed in June 2026. The study was conducted in accordance with the Strengthening the Reporting of Observational Studies in Epidemiology (STROBE) guidelines [15]. The present analysis reports sex-stratified findings from the same primary care cohort described in previous publications from this series [12,13,14]. The prior publication most closely related to the present work [12] examined overall, non-sex-stratified associations between physical activity, LDL-C, and TUG performance in the same cohort. The present study constitutes a distinct analytical contribution: it introduces sex-stratified analyses, evaluates sex as a potential effect modifier using formal interaction testing (PA × sex), and reports sex-stratified multivariable regression models—none of which were performed in the prior publication. Overlap in the description of the study setting, population, and data collection procedures reflects transparent reporting within a publication series and does not represent duplication of scientific content.

### 2.2. Study Population

A total of 863 adult patients aged ≥18 years were included. Inclusion criteria were: age ≥ 18 years, available PA classification, available LDL-C and TUG measurements, and complete core clinical variables. Patients with incomplete records were excluded. No pre-selection was performed based on sex, PA level, or cardiometabolic risk profile.

### 2.3. Data Collection

Data were obtained from electronic medical records as part of routine clinical assessment. All data were anonymized before extraction for research purposes and no patient identifiers were retained in the analytical dataset. Clinical variables included age, sex, BMI, LDL-C concentration, arterial hypertension (AH) status, and diabetes mellitus (DM) status. AH was defined as a documented clinical diagnosis and/or ongoing antihypertensive treatment. DM was defined as a documented clinical diagnosis and/or ongoing glucose-lowering treatment. LDL-C was measured using an enzymatic colorimetric assay in certified laboratories.

### 2.4. Physical Activity Assessment

PA was classified on the basis of patient self-report obtained during standard primary care consultations. Based on World Health Organization recommendations, PA was categorized as low (<150 min/week), moderate (150–300 min/week), or high (>300 min/week) [16]. In routine primary care, structured PA questionnaires or objective monitoring devices (e.g., accelerometers) are seldom feasible within standard consultation time. Brief verbal self-report categorized against established reference thresholds—consistent with the WHO Global Physical Activity Questionnaire (GPAQ) framework [17]—represents a pragmatic and clinically applicable approach for population-level PA stratification. Nonetheless, the absence of a validated instrument introduces potential recall bias and exposure misclassification, which should be considered when interpreting the findings.

### 2.5. Outcome Measures

The primary outcomes were LDL-C concentration (mmol/L) and TUG functional performance (seconds). TUG was assessed using a Garmin digital stopwatch (Garmin Ltd., Olathe, KS, USA) as part of routine primary care evaluation. Patients were instructed to rise from a standard chair, walk three meters, turn, walk back, and sit down. A TUG time ≥ 12 s was used as a clinically relevant threshold for impaired functional mobility [18].

### 2.6. Statistical Analysis

Statistical analyses were conducted using Python (version 3.11) with NumPy and SciPy libraries. Continuous variables are presented as mean ± SD. Categorical variables are presented as frequencies and percentages. Sex differences in baseline characteristics were assessed using independent samples *t*-tests for continuous variables and chi-square tests for categorical variables.

Sex-stratified associations between PA categories and LDL-C and TUG were assessed using one-way ANOVA followed by pairwise post hoc comparisons with Bonferroni correction. Multivariable linear regression models were constructed separately for males and females, with PA category entered as an ordinal variable (low = 1, moderate = 2, high = 3). Covariates in the LDL-C model were age, BMI, AH, and DM. Covariates in the TUG model additionally included LDL-C. To formally test for sex modification, an interaction term (PA × sex) was added to pooled models. Regression coefficients, 95% confidence intervals (CIs), and *p*-values are reported. Statistical significance was set at *p* < 0.05.

### 2.7. Ethical Considerations

This study was conducted in accordance with the Declaration of Helsinki (2013 revision). The analysis used fully anonymized retrospective clinical data collected during routine primary care practice. All data were fully anonymized before extraction for research purposes. No directly identifiable patient information was available during data extraction, statistical analysis, or manuscript preparation, and no patient identifiers were retained in the analytical dataset. According to Slovak legislation governing retrospective analyses of fully anonymized routinely collected clinical data, formal ethical committee approval and individual informed consent were not required. Patient consent was therefore waived.

## 3. Results

### 3.1. Baseline Characteristics

The study included 424 males (49.1%) and 439 females (50.9%). Baseline demographic and clinical characteristics are presented in Table 1. Males and females did not differ significantly in age, BMI, LDL-C, TUG, AH prevalence, DM prevalence, or PA category distribution (all *p* > 0.05). The cohort was well balanced between sexes across all measured variables.

### 3.2. LDL-C Across PA Categories by Sex

LDL-C concentrations across PA categories stratified by sex are presented in Table 2. In males, higher PA categories were associated with significantly lower LDL-C (ANOVA F(2421) = 13.03, *p* < 0.001). Post hoc comparisons demonstrated significantly lower LDL-C in the high PA group compared with both moderate PA (*p* = 0.003) and low PA groups (*p* < 0.001). In females, higher PA categories were also associated with significantly lower LDL-C (ANOVA F(2436) = 14.92, *p* < 0.001), with significant differences across all pairwise comparisons. Within each PA category, LDL-C did not differ significantly between males and females (all *p* > 0.05).

### 3.3. TUG Performance Across PA Categories by Sex

TUG performance across PA categories stratified by sex is presented in Table 3. In males, higher PA categories were associated with significantly lower TUG times (ANOVA F(2421) = 44.21, *p* < 0.001), with all pairwise comparisons reaching statistical significance after Bonferroni correction. In females, higher PA categories were also associated with significantly lower TUG times (ANOVA F(2436) = 36.09, *p* < 0.001), with all pairwise comparisons significant. Within each PA category, TUG times did not differ significantly between males and females (all *p* > 0.05).

### 3.4. Multivariable Regression—LDL-C

Results of the sex-stratified multivariable regression analyses for LDL-C are presented in Table 4. In both males and females, PA category was the only statistically significant independent predictor of LDL-C after adjustment (males: β = −0.301, 95% CI: −0.417 to −0.184, *p* < 0.001; females: β = −0.323, 95% CI: −0.440 to −0.205, *p* < 0.001). Age, BMI, AH, and DM were not independently associated with LDL-C in either sex (all *p* > 0.05). The overall model R^2^ was 0.060 in males and 0.069 in females. The interaction term (PA × sex) in the pooled model was not statistically significant (β = 0.021, *p* = 0.804), indicating no significant sex modification of the PA–LDL-C association.

### 3.5. Multivariable Regression—TUG

Results of the sex-stratified multivariable regression analyses for TUG are presented in Table 5. In both males and females, PA category was the strongest independent predictor of TUG performance (males: β = −1.235, 95% CI: −1.499 to −0.972, *p* < 0.001; females: β = −1.170, 95% CI: −1.456 to −0.885, *p* < 0.001). In females, age demonstrated a weak but statistically significant association with TUG (β = 0.011, *p* = 0.045). In males, DM was associated with higher TUG times (β = 0.509, *p* = 0.048). The overall model R^2^ was 0.186 in males and 0.151 in females. The interaction term (PA × sex) in the pooled model was not statistically significant (*p* = 0.944), indicating no significant sex modification of the PA–TUG association.

## 4. Discussion

### 4.1. Principal Findings

In this real-world primary care cohort of 863 adults, higher self-reported PA categories were associated with lower LDL-C concentrations and better TUG functional performance in both males and females. These associations were consistent in direction and magnitude across sexes, and formal interaction testing indicated no significant sex modification of either the PA–LDL-C or PA–TUG association. In multivariable regression, PA category was the strongest independent predictor of both outcomes in both sexes after adjustment for age, BMI, AH, and DM.

A key finding is that males and females in this cohort did not differ significantly in age, BMI, LDL-C, TUG, AH prevalence, DM prevalence, or PA distribution. This balanced baseline profile allowed for a meaningful sex-stratified comparison of PA–outcome associations without major confounding from differential baseline risk. The absence of sex modification suggests that the clinical relevance of PA assessment for cardiometabolic and functional health evaluation may be equally applicable to both male and female patients in primary care.

### 4.2. Comparison with Existing Literature

The present findings are broadly consistent with existing evidence demonstrating associations between PA and lipid profiles in both sexes [3,4]. Previous studies have reported sex-related differences in the magnitude of PA-related cardiovascular benefits, with some evidence suggesting that females may derive relatively greater cardiovascular mortality reductions from equivalent PA doses compared with males [7]. However, most of these studies focused on mortality endpoints and prospective designs rather than cross-sectional associations in primary care. The present findings extend the evidence base to a consecutive real-world primary care population using routinely available clinical variables. The consistency of PA–outcome associations across sexes, despite well-documented physiological sex differences, warrants further consideration. Male and female skeletal muscle differ in contractile properties, fiber-type composition, fatigue resistance, and hormonal milieu [10]. Sex-related divergences in neuromuscular performance characteristics have also been described [11]. The present findings suggest that, at the level of habitual PA classification in a primary care population, these intrinsic biological differences may not translate into meaningful sex modification of cardiometabolic or functional health associations. A plausible explanation is that the beneficial effects of regular physical activity—including reductions in sedentary behavior, improvements in metabolic efficiency, and maintenance of functional reserve—operate through pathways sufficiently robust to transcend underlying physiological sex differences at the population level. This interpretation is supported by the non-significant interaction terms for both PA–LDL-C (*p*  =  0.804) and PA–TUG (*p*  =  0.944) in the present cohort.

Regarding functional performance, evidence consistently demonstrates associations between PA and TUG performance across both sexes and age groups [5]. The present findings are consistent with this literature and further show that the PA–TUG association is equally prominent in males and females under real-world primary care conditions, with PA category explaining similar proportions of TUG variance in both sexes (R^2^ = 0.186 in males, 0.151 in females).

### 4.3. Clinical Implications

The consistency of PA–outcome associations across sexes supports the integration of structured PA assessment as a sex-independent component of cardiometabolic and functional health evaluation in primary care. The finding that PA was the dominant predictor of both LDL-C and TUG in both males and females—outperforming age, BMI, AH, and DM—underscores the potential clinical value of routine PA classification. It should be emphasized, however, that the self-report approach applied in the present study represents a pragmatic minimum for real-world primary care rather than a recommendation to forgo validated instruments. Validated tools such as the short-form International Physical Activity Questionnaire (IPAQ-SF) or the Godin Leisure-Time Exercise Questionnaire provide substantially richer and more reliable PA data, and their routine integration into primary care consultations should be encouraged where feasible. The present findings suggest that even a simplified PA categorization carries clinically meaningful signal, but this should be interpreted as a lower bound of what structured PA assessment can offer, not as an endorsement of non-validated approaches.

The present findings are consistent with the broader series of real-world cross-sectional analyses from this cohort, which demonstrated significant associations between PA and LDL-C [12], AH [13], and type 2 DM [14]. Taken together, this body of evidence supports PA assessment as a clinically relevant and sex-independent indicator across multiple cardiometabolic and functional health domains in primary care.

### 4.4. Limitations

Several limitations should be acknowledged. First, the cross-sectional design precludes causal inference and reverse causation cannot be excluded. Second, PA was assessed using self-report without a validated questionnaire or objective monitoring, introducing potential recall bias and exposure misclassification. Third, although sex-stratified regression models were adjusted for several clinically relevant covariates, residual confounding from unmeasured variables—including menopausal status, hormonal therapy, medications affecting lipids or functional performance, musculoskeletal conditions, and socioeconomic factors—cannot be excluded. Fourth, the study was conducted in a single primary care practice in Slovakia, which may limit generalizability. Finally, the relatively small high PA subgroups in sex-stratified analyses (males: n = 86, females: n = 79) may limit statistical power to detect small sex-specific differences. An additional limitation is the unavailability of data on lipid-lowering therapy, particularly statins. Statin use may reduce LDL-C concentrations independently of physical activity level, and its distribution may not be uniform across PA categories, potentially introducing residual confounding into the PA–LDL-C association that could not be controlled for in the present analysis. Future studies should incorporate detailed medication data to address this limitation.

### 4.5. Future Directions

Future studies should incorporate longitudinal designs and objective PA assessment to evaluate temporal and causal relationships between PA and cardiometabolic outcomes in sex-stratified primary care populations. Studies including menopausal status, hormonal therapy, and detailed medication data would provide a more complete picture of sex-specific PA–outcome relationships. Larger cohorts would improve statistical power for formal interaction testing and subgroup analyses.

## 5. Conclusions

In this real-world primary care cohort, higher self-reported PA was associated with lower LDL-C concentrations and better TUG functional performance in both males and females. These associations were consistent in direction and magnitude across sexes, with no statistically significant sex modification detected. PA category was the strongest independent predictor of both outcomes in both sexes after multivariable adjustment. These findings support the clinical relevance of routine PA assessment as a sex-independent indicator of cardiometabolic risk and functional health in primary care. Future longitudinal studies with objective PA assessment and more comprehensive covariate data are warranted.

## Figures and Tables

**Table 1:** Baseline demographic and clinical characteristics by sex. Continuous variables compared using independent samples *t*-test. Categorical variables compared using chi-square test. Abbreviations: AH, arterial hypertension; BMI, body mass index; DM, diabetes mellitus; LDL-C, low-density lipoprotein cholesterol; PA, physical activity; SD, standard deviation; TUG, Timed Up and Go.

Variable	Males (*n* = 424)	Females (*n* = 439)	Total (*n* = 863)	*p*-Value
Age (years), mean ± SD	51.4 ± 19.0	50.3 ± 19.6	50.9 ± 19.3	0.405
BMI (kg/m^2^), mean ± SD	27.6 ± 4.7	27.8 ± 4.5	27.7 ± 4.6	0.530
LDL-C (mmol/L), mean ± SD	3.53 ± 0.94	3.56 ± 0.94	3.54 ± 0.94	0.629
TUG (seconds), mean ± SD	10.18 ± 2.20	10.22 ± 2.31	10.20 ± 2.26	0.779
Arterial hypertension (AH), *n* (%)	239 (56.4%)	242 (55.1%)	481 (55.7%)	0.765
Diabetes mellitus (DM), *n* (%)	74 (17.5%)	74 (16.9%)	148 (17.2%)	0.887
Low PA, *n* (%)	170 (40.1%)	173 (39.4%)	343 (39.7%)	0.583
Moderate PA, *n* (%)	168 (39.6%)	187 (42.6%)	355 (41.1%)	
High PA, *n* (%)	86 (20.3%)	79 (18.0%)	165 (19.1%)	

**Table 2:** LDL-C concentrations across PA categories by sex. Post hoc comparisons performed using Bonferroni correction. * *p*-value for sex difference within each PA category (independent samples *t*-test). Abbreviations: LDL-C, low-density lipoprotein cholesterol; PA, physical activity; SD, standard deviation.

PA Category	*n*	Males LDL-C (mmol/L), Mean ± SD	*n*	Females LDL-C (mmol/L), Mean ± SD	*p* *
Low PA	170	3.74 ± 0.93	173	3.80 ± 1.00	0.514
Moderate PA	168	3.52 ± 0.95	187	3.51 ± 0.89	0.916
High PA	86	3.12 ± 0.81	79	3.14 ± 0.71	0.871
ANOVA *p*-value		*p* < 0.001 (F = 13.03)		*p* < 0.001 (F = 14.92)	

**Table 3:** TUG performance across PA categories by sex. Post hoc comparisons performed using Bonferroni correction. * *p*-value for sex difference within each PA category (independent samples *t*-test). Abbreviations: PA, physical activity; SD, standard deviation; TUG, Timed Up and Go.

PA Category	*n*	Males TUG (s), Mean ± SD	*n*	Females TUG (s), Mean ± SD	*p* *
Low PA	170	11.25 ± 2.16	173	11.20 ± 2.38	0.841
Moderate PA	168	9.72 ± 2.08	187	9.88 ± 2.14	0.451
High PA	86	8.96 ± 1.48	79	8.88 ± 1.51	0.718
ANOVA *p*-value		*p* < 0.001 (F = 44.21)		*p* < 0.001 (F = 36.09)	

**Table 4:** Sex-stratified multivariable linear regression for LDL-C. PA category entered as ordinal variable (low = 1, moderate = 2, high = 3). Models adjusted for age, BMI, AH, and DM. Interaction *p*: from pooled model with PA × sex interaction term. *** *p* < 0.001. Abbreviations: AH, arterial hypertension; BMI, body mass index; CI, confidence interval; DM, diabetes mellitus; LDL-C, low-density lipoprotein cholesterol; PA, physical activity.

Variable	Males β	Males 95% CI	Females β	Females 95% CI	Interaction *p*
PA category (per increase)	−0.301 ***	−0.417 to −0.184	−0.323 ***	−0.440 to −0.205	0.804
Age (years)	−0.001	−0.005 to 0.004	−0.003	−0.007 to 0.002	—
BMI (kg/m^2^)	−0.010	−0.029 to 0.008	0.002	−0.017 to 0.021	—
AH	0.005	−0.171 to 0.182	0.097	−0.075 to 0.268	—
DM	−0.079	−0.309 to 0.150	0.015	−0.213 to 0.243	—
**R^2^**	**0.060**		**0.069**		

**Table 5:** Sex-stratified multivariable linear regression for TUG performance. PA category entered as ordinal variable. Models adjusted for age, BMI, LDL-C, AH, and DM. Interaction *p*: from pooled model with PA × sex interaction term. * *p* < 0.05; *** *p* < 0.001. Interaction *p* for TUG: 0.944. Abbreviations: AH, arterial hypertension; BMI, body mass index; CI, confidence interval; DM, diabetes mellitus; LDL-C, low-density lipoprotein cholesterol; PA, physical activity; TUG, **Timed Up and Go**.

Variable	Males β	Males 95% CI	Females β	Females 95% CI	Interaction *p*
PA category (per increase)	−1.235 ***	−1.499 to −0.972	−1.170 ***	−1.456 to −0.885	0.944
Age (years)	0.006	−0.004 to 0.016	0.011 *	0.000 to 0.021	—
BMI (kg/m^2^)	−0.013	−0.054 to 0.028	0.004	−0.040 to 0.049	—
LDL-C (mmol/L)	−0.060	−0.271 to 0.150	0.086	−0.136 to 0.308	—
AH	0.368	−0.019 to 0.755	−0.189	−0.593 to 0.216	—
DM	0.509 *	0.005 to 1.012	0.098	−0.439 to 0.634	—
**R^2^**	**0.186**		**0.151**		

## Data Availability

The datasets used and/or analyzed during the current study are available from the corresponding author on reasonable request, subject to applicable ethical and data protection requirements.
